# Supplementary material for: The AGEs/RAGE Transduction Signaling Prompts IL-8/CXCR1/2-Mediated Interaction between Cancer-Associated Fibroblasts (CAFs) and Breast Cancer Cells
Source: Cells. 2022 Aug 4;11(15):2402. doi: 10.3390/cells11152402 (PMC9368521; doi:10.3390/cells11152402)
Supplement: Supplementary file 1 [file cells-11-02402-s001.zip › cells-1821937-supplementary.pdf]

Supplementary figures and figure legends

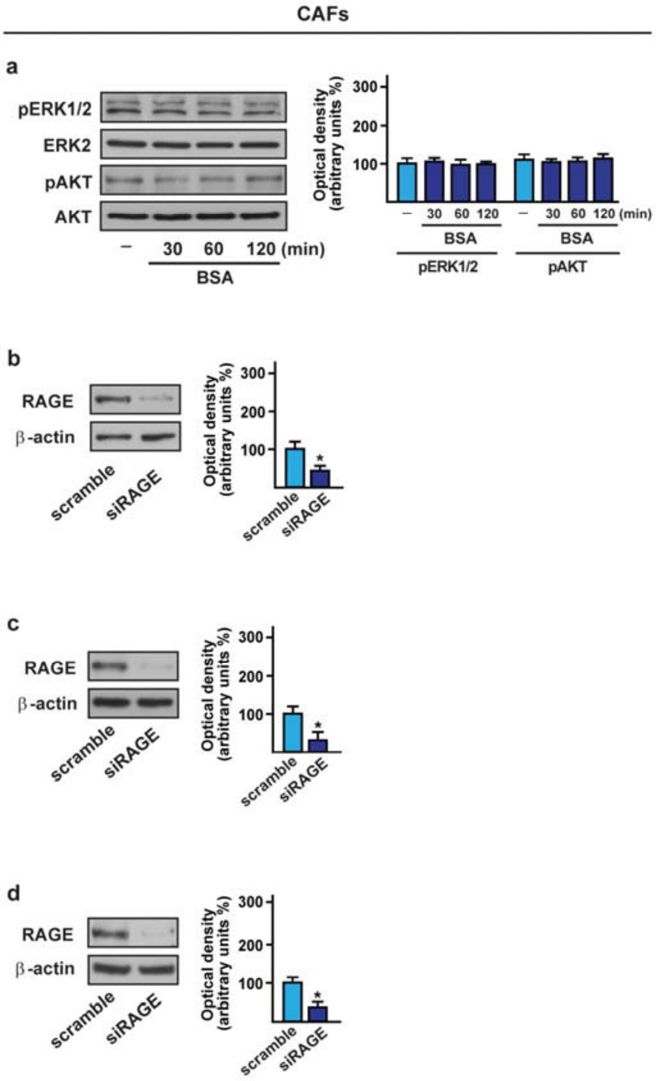

Figure S1. Phosphorylation of ERK1/2 and AKT upon BSA exposure and efficacy of RAGE silencing in CAFs. (**a**) Phosphorylation of ERK1/2 and AKT in CAFs treated with vehicle (-) and 100 μg/ml BSA for the indicated times. ERK2 and AKT served as loading control, as indicated. (**b**) Representative immunoblot showing the efficiency of RAGE silencing in CAFs. (**c**) Representative immunoblot showing the efficiency of RAGE silencing in CAFs. (**d**) Representative immunoblot showing the efficiency of RAGE silencing in CAFs. β-actin served as a loading control. Side panels show densitometric analysis of the blots normalized to the loading controls. Values represent the mean ± SD of three independent experiments. (*) indicates $p < 0.05$.

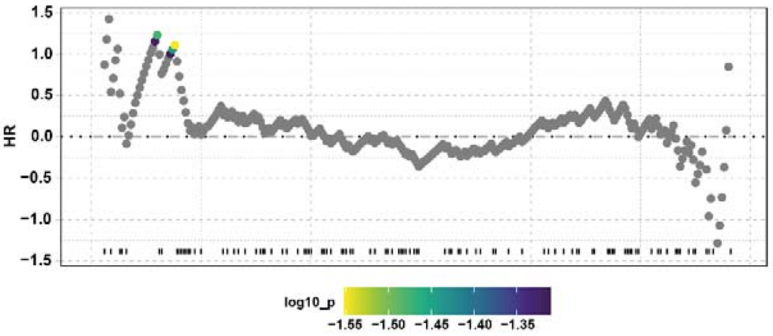

Figure S2. The plotALL function of the survivALL package calculating hazard ratios (HR) (y-axis) for all possible CXCR1/2 cut-points to be examined. Affymetrix basal breast cancer patients were ranked by the increasing expression of CXCR1/2 (x-axis). The color bar gradient indicates the range of the most significant points-of-separation of the population (low-high significance = blue-yellow gradient) based on CXCR1/2 expression and relapse free survival (RFS) of each patient.

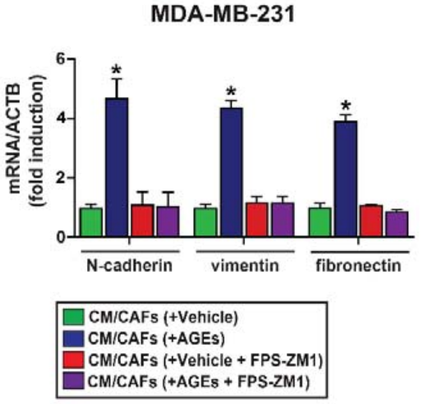

Figure S3. mRNA expression of N-cadherin, vimentin and fibronectin evaluated by real-time PCR in MDA-MB-231 exposed for 6 h to CM collected from CAFs previously treated with vehicle or 100 μg/ml AGEs alone and in the presence of 1 μM RAGE inhibitor FPS-ZM1. Values were normalized to the β actin (ACTB) expression and shown as fold changes of mRNA expression upon treatments compared to cells exposed to vehicle. Each column represents the mean ± SD of three independent experiments performed in triplicate. (*) indicates $p < 0.05$.
